# “If you have a pain, get on a plane”: qualitatively exploring how short-term Canadian international retirement migrants prepare to manage their health while abroad

**DOI:** 10.1186/s40794-021-00136-4

**Published:** 2021-04-12

**Authors:** John Pickering, Valorie A. Crooks, Jeremy Snyder, Trudie Milner

**Affiliations:** 1 Simon Fraser University, 8888 University Dr, Burnaby, BC V5A 1S6 Canada; 2 Yuma Regional Medical Center, 2400 S Avenue A, Yuma, AZ 85364 USA

**Keywords:** International retirement migration, Canada, United States, Travel health, Insurance, Preparation

## Abstract

**Background:**

Every year, tens of thousands of older Canadians travel abroad during the winter months to enjoy warmer destinations that offer social and recreational opportunities. How do these Canadians prepare to manage their health while abroad? In this analysis we explore this question by developing a typology of preparatory strategies.

**Methods:**

Semi-structured interviews were conducted with 19 older Canadians living seasonally in Yuma, Arizona (United States). Interviews were transcribed verbatim and thematically analysed to form the basis of a typology of preparatory strategies.

**Results:**

Four distinct preparatory strategies form the typology that summarizes how Canadian international retirement migrants prepare to manage their health while abroad. First, some participants became thoroughly prepared by gathering information from multiple sources and undertaking specific preparatory activities (e.g., visiting a travel medicine clinic, purchasing travel health insurance, bringing prescription refills). Second, some participants were preparation-adverse and relied on their abilities to address health needs and crises in-the-moment. Third, some participants became well informed about things they could do in advance to protect their health while abroad (e.g., purchasing travel health insurance) but opted not to undertake preparatory actions. A final group of participants prepared haphazardly.

**Conclusions:**

This typology can assist health care providers in international retirement migrant destinations to appreciate differences among this patient population that is often characterized as being relatively homogenous. More research is needed to determine if these preparatory strategies are common in other mobile populations and if they are found in other destinations popular with international retirement migrants.

## Introduction

Although research exists regarding strategic health behaviours and decisions among international travellers [1–5], there has been little consideration of these issues among short-term or seasonal international retirement migrants. These migrants are older persons, typically over the age of 60, who travel abroad for the winter season or for short periods in order to enjoy the benefits of destinations with warmer climates that provide social, recreational, and even health-promoting opportunities [6–8]. International retirement migration is a widespread global practice, and Canadians are among those who participate in this short-term transnational mobility. Marshall et al. [9] identified some personal health management strategies undertaken by older Canadians living seasonally in Florida (United States [US]), which include pre-filling prescriptions prior to departure, purchasing travel health insurance, and maintaining a schedule of health check-ups with their family doctor prior to going abroad. Generally, however, little is known about the individual-level decision-making and action-taking choices these older Canadian travellers make in order to prepare to manage their health while abroad for weeks or months at a time. Meanwhile, it is important that we be attentive to such preparatory practices given that it is common for international retirement migrants to be managing multiple chronic health conditions that they will want to avoid exacerbating while abroad and that they are also at an age where the risk of acute health events (e.g., stroke, heart attack) that may require urgent medical attention is more pronounced [10–13].

The Canadian experience of seasonal migration presents its own challenges that likely shape if and how these travellers prepare to manage their health while abroad. This is particularly noticeable in issues surrounding paying for and accessing health care elsewhere, as well as in related stressors such as accessing health records while abroad. For example, seasonal retirement migrants from Canada to the US must ensure they spend more than 6 months of the year in their Canadian home province or territory to maintain their public health care health coverage. These same Canadians need to also manage a complex, multi-year averaging of time spent in the US to avoid requirements to pay US income tax on worldwide earnings [14, 15]. At home, Canadians have access to universal health care that is provided with no fee at the point-of-service for medically necessary care and is funded publicly through taxation. This universal access, however, is not portable to other countries when Canadians are travelling abroad [16, 17]. Canadian travellers who want to have health insurance coverage while abroad need to privately purchase travel health insurance, and this is clearly only an option for those who can afford such costs [9, 18]. While the insurance industry is federally regulated in Canada, there are significant differences in cost and coverage between companies [19]. This variation creates a complex and convoluted network of providers that Canadian international retirement migrants must navigate if they want to purchase a travel health plan. There is no legal requirement to have private health insurance while abroad and so there are many Canadians – including short-term international retirement migrants – who opt not to purchase coverage [20].

How can we characterize the preparatory strategy or strategies that older Canadians employ prior to going abroad as international retirement migrants to manage a health need or crisis? To answer this question, we developed a qualitative study based on semi-structured interviews with Canadian retirement migrants living seasonally in Yuma, Arizona. Due to its warm and dry climate, Yuma is a popular destination for both domestic and international retirement migrants in the US [21, 22] estimated the city’s year-round population to be just over 100,000; however, Yuma is not a wintertime destination filled with retirees’ yachts and mansions. Instead, many retirement migrants who winter in Yuma do so in trailers that they drive in or that are permanently affixed, congregating together in the various gated lifestyle communities throughout the city or rough camping in the surrounding desert areas. Despite the number of retired migrants being untracked, untraced and unregulated, numerous unsubstantiated estimates of their numbers abound on internet sites. These sites are purposefully designed to inform retirement migrants and often claim the population of Yuma doubles during the winter months with an additional 100,000 seasonal residents, many thousands of whom are thought to be Canadian retirees (e.g., [23, 24]).

In this analysis we explore how Canadian international retirement migrants wintering in Yuma prepared to manage their health while abroad, including the steps they took while still in Canada and any planning undertaken to access health care while in the US. Through thematic analysis of the interview findings we identify a typology of preparatory strategies, which is presented herein. We detail these strategies and then move to situate our findings within the context of the challenges surrounding the seasonal migration of older Canadians and the existing travel health and medicine literatures. This analysis is not only a novel contribution to the growing literature on short-term or seasonal international retirement migration (see [25] for an overview), but the typology is an important informational tool that can be used by health care providers and administrators in destinations to understand some important differences among this seemingly relatively homogenous patient group [20, 26]. Our own recent research in Yuma, for example, has shown that providers and administrators there have little familiarity with the Canadian health care system or differences among Canadian retirement migrants with regard to private health insurance coverage and entitlements [21]. Having awareness of these different approaches to preparation can also be beneficial in the Canadian context of health care provision. For example, the typology presented herein can assist Canadian international retirement migrants’ family physicians with better understanding the diverse educational needs among this patient group. The range of preparatory strategies documented in the typology underscores the importance family physicians proactively discussing factors such as prescription renewals, health care access, and health management with their older patients who take part in this transnational mobility. Doing so is consistent with family physicians’ roles in supporting pre-travel consultations that include education proactively managing health risks [27].

## Methods

A qualitative case study approach was used to explore the health management experiences of Canadian short-term international retirement migrants wintering in the popular destination of Yuma, Arizona. Consistent with case study methodology [28, 29] and building on previous studies in Yuma [25], we drew on multiple sources of information to understand important contextual aspects of this city as a destination for Canadian retirees. In addition to conducting face-to-face interviews, our information sources included touring health and social care sites in the city, reviewing publicly available information about Yuma as a destination for retirement migrants, and observing aspects of everyday life in Yuma during our fieldwork. Our observations involved on-site interactions with facilities that older persons frequent, as well as infrastructures targeting seniors. While this analysis is focused on the findings specific to the interviews, this wider observational information was integral to the manner in which both data collection and analysis was conceptualised.

### Recruitment

Following approval from our institutional research ethics board, participant recruitment began. Given the exploratory nature of this study, our goal was to recruit up to 20 Canadian international retirement migrants living seasonally in Yuma as participants for one-on-one interviews during our on-site fieldwork. After some requests to do so, we agreed to allowing some small group interviews to occur for participants who preferred to be interviewed with a partner or friend. After piloting a number of recruitment strategies (e.g., social media postings, advertisements on community billboards), one strategy proved most successful. Specifically, this involved placing a postcard with study details and contact information on the windows of vehicles with Canadian license plates parked at malls, grocery stores, gyms, clothing retailers, restaurants, and other such establishments. Recruitment was guided by a temporal cut-off in that interviews took place during our on-site fieldwork in January, 2018. While having a temporal cut-off can limit sample size, in the case of the current study this was significantly offset by the benefits of in-situ research, including the ability to facilitate the trust-building associated with interviewing. Prospective participants who responded to the invitation by email were sent a follow-up email which included a letter of invitation containing study details, its purpose, proposed interview dates, and information on how to participate. Interested participants were asked to reply to the lead investigator by e-mail to confirm eligibility (i.e., a Canadian living seasonally in Yuma over the age of 60) prior to scheduling the interview.

### Data collection

All interviews were held in January 2018 at a place of the participant’s choosing and ran for approximately 90 min. To enhance consistency, all interviews were conducted by a single member of the research team. They were recorded digitally and later transcribed verbatim. Recording started after a review of the study details and completion of a signed consent form. A semi-structured interview guide was used to guide the conversations. This guide was created following a review of relevant literature and through a process of confirmation among the investigators. Questions in the guide probed: why participants chose Yuma as their winter destination; personal health; health care-related preparation prior to departing Canada; experiences with accessing health care while in Yuma, and informational continuity of care (e.g., health records, prescription lists). Consistent with a semi-structured approach, participants were invited to touch on topics of discussion not raised in the interview guide that they thought were relevant to the discussion. Upon completion of the interviews, participants were given a US$10 gift card to a local coffee shop to acknowledge their valuable contributions to the study.

### Analysis

In preparation for coding and thematic analysis, verbatim interview transcripts were independently reviewed by all investigators. After the independent reviews, team meetings were arranged to identify emergent themes by contrasting the issues discussed by participants against the existing literature and the contextual insights gleaned from first-hand observations while in Yuma. Through this analytic process we arrived at developing a typology that characterizes the ways in which Canadian international retirement migrants approach preparing to manage their health while abroad. The typology characterizes four distinct, nuanced strategies used for such preparation. After developing this typology as an analytic framework, a coding structure was created that captured themes central to each preparation strategy. The first author conducted the coding, which was done via a highlighting and extracting system in a word processing program. The second author assisted with resolving any coding uncertainties. Coded extracts were shared with the investigators to seek confirmation on interpretation of the codes and the overall typology. The use of investigator triangulation throughout the analytic process, the establishment of an audit trail by keeping a record of important decisions, and the inclusion of direct quotes in the following section to build trustworthiness are all factors that contribute to the rigour of this analysis (e.g., [30–32]).

## Results

We interviewed 19 Canadian international retirement migrants wintering in Yuma, Arizona, most of whom were women (*n* = 12). The majority of participants had spent multiple winter seasons in Yuma, and some had visited other popular retirement migrant destinations in the US and Mexico. Participants ranged in age from 63 to 86 and had travelled from several Canadian provinces, including: British Columbia (*n* = 8), Alberta (*n* = 6), Manitoba (*n* = 2), Saskatchewan (*n* = 2), and Yukon (*n* = 1). While most considered themselves to be in good health overall, many had experienced health exacerbations while abroad ranging from developing influenza to requiring hospitalization and ultimately repatriation due to diverticulitis. Participants drew on their deep lived experiences of this transnational practice during the interviews to reflect on how they managed their health while abroad and their plans for doing so prior to departing for Yuma. These plans included actions such as preparing to access health care before departure from and upon return home to Canada, accessing health care while in Yuma, filling prescriptions in advance or planning for renewals while abroad, purchasing travel health insurance, making copies of health records, and undertaking other similar activities that may facilitate care continuity and health management in this transnational context.

Through independent and triangulated transcript review and thematic analysis we identified four distinct preparatory strategies that together form a typology of how Canadian international retirement migrants planned for managing their health while abroad. First, there were those who chose to become thoroughly prepared for a range of potential outcomes by undertaking extensive, and often well-informed, research and preparatory activities. Second, some participants were highly preparation-adverse and opted to not prepare at all for the possibility of needing to access health care or have to take on any active health management while abroad. A third group characterized themselves as well informed, and as a result of what they had learned had opted to not undertake any preparatory actions that required financial investment (e.g., purchasing travel health insurance). Finally, a fourth group prepared in a haphazard way, often acting on information and advice that was obtained in a non-systematic way. In the remainder of this section we expand on these four distinct preparatory strategies, drawing on verbatim quotes from participants to support interpretation.

### Becoming thoroughly prepared

Participants who characterized themselves as thoroughly prepared for a range of potential health and health care access outcomes while abroad relied upon extensive information gathering prior to, and during, their time away. In particular, they typically spent numerous hours researching different options for travel health insurance, often obtaining multiple quotes and speaking with a range of brokers. For example, as one participant explained “…*we had Manulife recommended to us [by friends] and we thought, ‘well it’s a big company.’ So, we checked them out and decided to try it out and they were absolutely great*.” Word-of-mouth was an important way to learn about travel health insurance plans for those wanting to prepare for their time abroad in such a way. Another participant described changing travel health insurance providers “…*after we’d heard so many complaints on [provider] about rejecting [health] claims*” from other retirement migrants. Others’ experiential knowledge also informed many participants’ decisions regarding where to access prescriptions while abroad. For example, participants received advice on whether or not buying pharmaceuticals across the border in Mexico (a very short drive from Yuma) was thought to be a safe option, or whether or not prescriptions should be filled in Canada before departing for the US. Participants’ own histories as international retirement migrants also shaped the preparatory activities they undertook. For example, while in Canada prescriptions cannot often be written for longer than a two- or three-month supply of a drug, some participants had identified creative solutions: *“I need six months [worth of prescriptions] … So, I called the doctor [before departure], and said, ‘I want you to double the dosage.’ She said, ‘I’ll give you prescription for twice a day’ and I just take it once.”*

The preparatory planning of thoroughly prepared participants extended into the period after returning to Canada from Yuma. *“Your annual physical, you get ‘em done as soon as you go home, you get all the doctor stuff out of the way when you get home, because you need six months clear when you return.”* Signaled by this quote, many travel health insurance providers required a period of many months with no major health episodes or new prescriptions in order to issue policies for future travel. Thoroughly prepared travellers thus planned in advance to receive their annual physical exam from their family physicians shortly after returning home from Yuma so that they would have the health history record needed to purchase a travel medicine policy for the next winter season. From when to get their flu shot to when to start calling potential travel medicine insurers, and everything in between, we learned that many thoroughly prepared participants diarized specific preparatory activities so that they could create a sound plan for managing their health while abroad and act in the event they needed to access health care while in Yuma. These planned and diarized activities also extended to health-related events including attending free screenings and educational workshops, known as the Silver Care program, offered by the Yuma Regional Medical Center.

### Being preparation-adverse

Those who were preparation-adverse actively avoided preparing for the potential of having to manage health-related issues while living abroad for the winter. Although there were a variety of reasons given for why some participants were preparation-adverse, they all focused on a desire to not have to plan for some of the complexities of transnational living prior to arriving in Yuma. In some instances, participants lacked technological skills and were not confident in their abilities to get details from websites and apps, which they explained were key informational platforms for preparatory strategies (e.g., cost comparing travel health policies, reading information in online forums, looking up details of health service availability in Yuma). Instead, they opted to not seek out information or prepare for eventualities. In other instances, some of those who were preparation-adverse firmly believed that if they developed an acute or exacerbated chronic health condition, they would be stable enough to return home without needing to access care in Yuma. As one participant explained: *“My goal is to just try to stay healthy down here and not need any healthcare, because really I’d rather deal with it at home.”* Others still had a firm belief that if they needed prescriptions refilled, had an emergency situation develop that required going to the hospital, or required some other form of medical intervention they would be able to easily make arrangements to do so while in Yuma without preparing in advance.

Unlike their more prepared counterparts, preparation-adverse participants did not spend time researching or purchasing travel health insurance prior to arriving in Yuma, nor did they worry about having checkups or physicals with their physicians at home prior to travel or upon return to Canada. These choices were often presented in a manner that suggested participants were resigned to the fact that travel health insurance would either be too costly, or that they would be ineligible. *“Some of us [Canadian international retirement migrants] do come without it [travel health insurance] … If you’ve got all kinds of health issues, it’s gonna cost you more and they just figure they’ll go take the chance.”* For those for whom travel health insurance was too costly, the belief was: *“If you have a pain, get on a plane.”* Interestingly, many preparation-adverse participants discussed the importance of using their social networks as a buffer against the potential negative consequences of their lack of preparation. For example, some believed that they could rely on other members of their residential communities in Yuma to offer recommendations for where to purchase pharmaceuticals, advise them on regulatory matters regarding accessing emergency medical care without travel health insurance, and the like.

### Preparing by becoming informed yet not taking action

Unlike those who were thoroughly prepared to manage their health while in Yuma for the winter, there was another group of participants who had become well informed about a range of health matters but had chosen not to undertake any specific preparatory actions. Participants who undertook this strategy were generally knowledgeable about things such as travel health insurance, when and where to get prescription refills, and health care facilities in Yuma, but did not use this knowledge to guide any preparatory actions. Such research led many of those who opted not to purchase a policy to find the application process to be complex and criteria for approval to be restrictive, which is why this particular preparatory action was not adopted. As one participant explained: “*They [insurance providers] do that [make the approval process complex] on purpose so they can screw you later*” in terms of the lack of clarity regarding which pre-existing conditions may lead to non-coverage in the case of medical treatment while abroad. Phrases such as “*scam*” and “*cartel*” were commonly used by these participants when describing what they had learned about the travel insurance sector and policy options. Participants in this group shared a commonly held belief that unfortunate events would happen to other people, but not themselves, and used such beliefs to inform decisions regarding not purchasing travel health insurance.

The tendency to become informed, yet avoid taking action, was motivated by a strong sense of self-reliance. This strategy resulted in these participants’ conscious decisions to avoid taking actions that others, and especially those who were thoroughly prepared, identified as beneficial. For example, despite knowing the benefits of regular visits with their doctor after returning home, many simply chose not to: *“No we don’t [visit our doctor after returning home]. Why would we? Our meds aren’t going to change.”* Another element of self-reliance was these participants’ common believe that they were “*in good enough health”* to take the risk of opting not to purchase travel medicine insurance for their time in Yuma, despite having done some research into the options available.

### Becoming haphazardly prepared

A haphazard preparatory strategy was employed by participants who did not take a systematic or comprehensive approach to informing their decisions regarding which preparatory activities to undertake, or who undertook preparatory actions without becoming extensively informed. Much of their information gathering was done in situ through talking with friends and neighbours while already abroad. As one participant explained: *“I mean I think that’s how snowbirds operate a bit. It’s, it’s so much as word of mouth.”* While thoroughly prepared participants also cited the importance of experiential knowledge as an information gathering and knowledge building tool, those who prepared haphazardly relied on it as their primary source and so only received information that others chose to share. Because much information was gathered once having arrived in the destination, in many cases critical preparatory steps were missed. For example, some participants discussed carrying copies of recent medical records with them to Yuma, but not a full list of their prescriptions. In another instance, a participant had a complete list of their medications, but only learned of the advantages of having their annual physical exam with their family doctor upon return to Canada through word-of-mouth.

As with most other participants who had purchased travel health insurance, those who prepared haphazardly typically hoped they would not need to use their policies. *“We’re kinda desperate not to use it [travel health insurance]. We pay all this money for it in case you’re in a car accident or have something major.”* There were also those who opted not to purchase travel health insurance at all. Many haphazardly prepared individuals reported learning through their social networks about the seemingly affordable pharmaceuticals and medical care available just across the border in Los Algodones, Mexico. They viewed it as a reliable alternative to using travel health insurance policies:

*We don't have extended health care; we don't even have a pension. We, neither one of us, all we have is our [national contributory pension] and, well she gets [national old age security pension]. I don't get that yet. And so, we don't have pensions, we don't have extended health plan, so we do go to [Los] Algodones.*

By relying heavily on others in their residential communities for health-related information in lieu of doing their own information gathering, in many ways these participants were also listening to others’ risk assessments regarding accessing care and purchasing pharmaceuticals in Los Algodones.

## Discussion

Each year a significant number of retired Canadians travel abroad to live seasonally in warmer climates. Our analysis of interviews with Canadians who travel to a specific destination in the US has identified four distinct preparatory strategies employed by these migrants to manage their health while living abroad seasonally. Each strategy is comprised of two common components, which are: (1) information gathering, and (2) preparatory actions. Table 1 provides a synthesis of these strategies and identifies specific examples of information gathering and preparatory actions. It is important to note that there are some intersections or points of commonality between strategies. For example, “thoroughly prepared” and “haphazardly prepared” strategies both produce knowledgeable individuals who also undertake a number of preparatory actions. Information sources and specific actions vary between these two groups, though. Thoroughly prepared individuals incorporate the experiential knowledge of others into their overall body of knowledge, while those who haphazardly prepare rely on it almost exclusively. This is an important distinction that ultimately has a profound impact on the knowledge systems international retirement migrants draw on to make (seemingly) informed decisions and put knowledge into action. In the remainder of this section we consider the findings in light of the existing knowledge base, identify their implications for health services providers who care for international retirement migrants while abroad, and consider directions for future research.

**Table 1:** Synthesis of Canadian international retirement migrants’ preparatory strategies for managing their health while abroad

	*Scope of the strategy*	*Examples of information gathering strategies*	*Examples of preparatory actions taken*
*Thoroughly prepared*	an international retirement migrant who carefully plans how they will manage their health abroad and undertakes preparatory actions based on this plan	undertakes multiple strategic searches for travel health insurance information; researches health-related events for the winter season	schedules full annual medical examination upon return to Canada; travels with pre-filled prescriptions for the duration of the stay in the US
*Preparation adverse*	an international retirement migrant who undertakes little-to-no advanced planning and intends to act reactionarily to any health events or issues that emerge while abroad	receives word-of-mouth details about health management both home and abroad	plans to call on friends and acquaintances to provide financial support needed; plans to fly or drive home if health complications arise
*Prepared with info but not action*	an international retirement migrant who consults multiple information sources, considers the types of health management issues they may encounter while abroad, and then opts not to prepare in advance	researches travel health insurance options	does not take preparatory actions
*Haphazardly* *prepared*	an international retirement migrant who is generally knowledgeable about health management, but has knowledge gaps and a non-systematic approach to gathering information that may result in taking risks	relies on incomplete information shared by friends and acquaintances	looks for affordable prescription refills while abroad; plans to avoid making travel health insurance claims

Existing research demonstrates that it is common for many types of travellers to be unprepared to manage potential health events while abroad [33, 34], and some evidence has emerged that older travellers tend to be less prepared than younger ones [35]. These issues are echoed in this study, which has shown that there are Canadian international retirement migrants who are similarly unprepared. These failings in preparation, that have also been documented elsewhere, include non-existent or inadequate travel health insurance coverage [36, 37], poor prescription medication management [10, 38], incomplete documentation of past medical history [39], as well as limited knowledge of sources to connect with if problems do occur [40]. In the context of the current study, these gaps in preparation are very concerning given that migrants’ ages may very well amplify the associated risks. More specifically, Canadian international retirement migrants have comparatively higher risks of experiencing negative health events while abroad than the average traveller due to their age, which corresponds to an elevated risk of numerous health complications [10–12]. As expected, many participants were aware of these risks and opted to become well-prepared and informed in an effort to mitigate negative outcomes. However, our findings also show that knowledge of risks does not always translate into undertaking preparatory actions. Evidence of inaction heightens the importance of public health practitioners and physicians providing travel health education to international retirement migrants [41, 42]. Our findings underscore the need for travel medicine interventions that target international retirement migrants to ensure they can make informed decisions regarding how best to manage their health while abroad [27]. The Canadian Committee to Advise on Tropical Medicine and Travel could, for example, begin to identify best practices to support the development of interventions such as informational tools [43].

Much of the existing literature on short-term and seasonal international retirement migration suggests these travellers share socio-economic similarities [44, 45], particularly given that they all require the financial means to travel and live abroad for extended periods and maintain a residence at home and abroad. While looking at a more granular aspect of the international retirement migration experience in the context of the current study, we found important differences emerged among retired Canadians who traveled to Arizona for the winter. Specifically, differences became clear in how the interview participants prepared for, and perceived, potential health risks in relation to accessing health care and managing their health in a transnational context. For those who aim to improve the travel health awareness and literacy of international retirement migrants, these differences point to the fact that multiple types of interventions (e.g., informational tools, conversations with family physicians, travel health insurance purchase navigational aids) are likely needed to target all four of the traveller types identified in our analysis in order to address their associated concerns and comfort with advanced preparation. This is consistent with the approach advocated for by Suh and Flaherty [46], who suggest that older travellers benefit from tailored approaches to improving their travel medicine awareness and overall travel health. Many of our interview participants demonstrated particular distrust of, and a poor understanding of, the travel health insurance industry. Other research has documented similar concerns about insurance providers and lack of preparatory practices among individuals travelling regularly for business purposes, migrant farm workers, immigrant travellers visiting friends and relatives (e.g., [33, 47]). Identifying ways to assist Canadian international retirement migrants with making *informed* decisions about travel health insurance options is a clear need that emerges from the current study. Our finding that Canadian international retirement migrants are not a homogenous group when it comes to preparing to manage their health while abroad is also an important learning point for members of the travel insurance industry, in addition to health care providers in destinations who may assume that Canadian travellers have mostly participated in similar preparatory activities around insurance acquisition or prescription renewals [21, 48].

The findings shared in the previous section hold a number of implications for further research, four of which we highlight here. First, in the current study we did not explore the factors and constraints that shaped decision-making around preparatory strategies. Now that the typology presented herein has been formed, future research should explore deeper understandings of why particular preparatory strategies are adopted by specific Canadian international retirement migrants and their links to factors such as access to income, personal values and beliefs, and internet competency. Second, as was apparent from these interviews, widespread distrust towards the travel health insurance industry among Canadian international retirement migrants exists. Unfortunately, there is a paucity of research focused on most dimensions of travel health insurance, including trust-building with and decision-making by older travellers. We view this as an area worthy of further research consideration so that this information can assist with identifying ways to support informed decision-making not just about travel health insurance, but about all facets of travel medicine by international retirement migrants. Third, similar research undertaken in other US and international destinations popular with Canadian international retirement migrants will assist with shedding light on whether or not any destination-specific factors shape the preparatory actions or information gathering strategies undertaken by Canadian international retirement migrants to manage their health while abroad. Finally, a number of people other than the traveller themselves are involved in supporting the ways in which Canadian international retirement migrants prepare for managing their health while abroad through information gathering and key actions. Among those mentioned in the findings are Canadian family physicians and pharmacists. It would be very useful to undertake research that captures the professional perspectives held by these groups regarding the opportunities and challenges they encounter in supporting these patients in their travel plans as well as their insights on ways to support informed decision-making that can secure the best health outcomes.

As with all research, there are some limitations associated with this study that we must acknowledge. First, our recruitment materials and interviews were available only in English, and thus those Canadian international retirement migrants in Yuma who were not fluent in English were not able to participate. Second, our strategy of placing recruitment information on vehicles with Canadian license plates excluded Canadian international retirement migrants who drive owned or rented vehicles registered in the US while in Yuma. Third, as we noted in the methods section, we adopted a temporal cut-off for data collection. Although we previously justified our use of this approach, we do acknowledge that it limited our ability to identify specific participant gaps in the sample and target their recruitment. Outside of these limitations, there are other important considerations that are worthy of being addressed. For example, because there is not reliable tracking of Canadian international retirement migrants at the population-level, we cannot know how common or uncommon Yuma is as a destination for Canadians nor the sex distribution of Canadians who stay there. Because of this, we simply cannot know whether the sex distribution of 12 women and 7 men found in our study is representative. However, as this is a qualitative study, we have not sought to achieve representativeness in the sample and thus this does not serve as a true limitation but is a consideration worthy of being acknowledged. Similarly, because of our qualitative design our study is exploratory and we do not seek generalizability. Instead, we seek transferability through sharing important contextual aspects of the case study that will enable others to determine whether or not the findings may have relevance to other contexts.

## Conclusion

This exploratory, qualitative analysis found Canadian seasonal international retirement migrants engage in a variety of preparatory practices to manage their health during their time spent living abroad. Specifically, we identified four distinct preparatory strategies composed of both approaches to information gathering and preparatory actions undertaken. First, some participants became thoroughly prepared through gathering information from multiple sources and undertaking various preparatory activities (e.g., researching material online, purchasing travel health insurance, obtaining prescriptions for the duration of stay). Second, some participants were preparation-adverse and chose reactionary strategies to manage emerging health issues. Third, some participants became well informed in advance to protect their health while abroad, such as purchasing travel health insurance, but opted to avoid undertaking preparatory actions. A final group of participants prepared haphazardly. These findings support lessening reliance on one-size-fits-all approaches to health care intervention strategies (e.g. medical outreach programs, free health screenings, etc.) and informational campaigns for Canadian international retirement migrants.

Growing research attention is being given to aging, reflecting the upward trends in population aging in the Global North in particular (e.g., [49, 50]). Much of this research is fixed-in-place or centres on mobility limitations or immobility, which is evident in studies on aging-in-place (e.g., [51–54]), and assistive transportation (e.g., [55, 56]). In the current study, we shifted this narrative by having focused on a transnational mobility practiced by some older Canadians and others internationally, thereby focusing on older people as living mobile lives. International retirement migrants need to manage tax and health system requirements both at home and abroad, which may require carefully navigating complex requirements (e.g., [14, 17, 57]). As this analysis showed, they must also make decisions regarding how they will manage their health while abroad that can involve seeking out information and undertaking preparatory actions while at home and away. Aging can be a mobile practice and studies such as the current one can help to identify the policy and practical implications of older people’s transnational engagements, which are thus an important complement to work that frames aging as relatively fixed-in-place or localized.

## Data Availability

Due to privacy concerns for participants, the data for this study is not publicly available.
